# Reliability in evaluator-based tests: using simulation-constructed models to determine contextually relevant agreement thresholds

**DOI:** 10.1186/s12874-018-0606-7

**Published:** 2018-11-19

**Authors:** Dylan T. Beckler, Zachary C. Thumser, Jonathon S. Schofield, Paul D. Marasco

**Affiliations:** Laboratory for Bionic Integration, Department of Biomedical Engineering, ND20, Cleveland Clinic, 9500 Euclid Avenue, Cleveland, OH 44195 USA

**Keywords:** Inter-rater, Inter-evaluator, Reliability, Agreement, Krippendorff’s alpha, Index of reliability, Intercoder, Interrater, Threshold

## Abstract

**Background:**

Indices of inter-evaluator reliability are used in many fields such as computational linguistics, psychology, and medical science; however, the interpretation of resulting values and determination of appropriate thresholds lack context and are often guided only by arbitrary “rules of thumb” or simply not addressed at all. Our goal for this work was to develop a method for determining the relationship between inter-evaluator agreement and error to facilitate meaningful interpretation of values, thresholds, and reliability.

**Methods:**

Three expert human evaluators completed a video analysis task, and averaged their results together to create a reference dataset of 300 time measurements. We simulated unique combinations of systematic error and random error onto the reference dataset to generate 4900 new hypothetical evaluators (each with 300 time measurements). The systematic errors and random errors made by the hypothetical evaluator population were approximated as the mean and variance of a normally-distributed error signal. Calculating the error (using percent error) and inter-evaluator agreement (using Krippendorff’s alpha) between each hypothetical evaluator and the reference dataset allowed us to establish a mathematical model and value envelope of the worst possible percent error for any given amount of agreement.

**Results:**

We used the relationship between inter-evaluator agreement and error to make an informed judgment of an acceptable threshold for Krippendorff’s alpha within the context of our specific test. To demonstrate the utility of our modeling approach, we calculated the percent error and Krippendorff’s alpha between the reference dataset and a new cohort of trained human evaluators and used our contextually-derived Krippendorff’s alpha threshold as a gauge of evaluator quality. Although all evaluators had relatively high agreement (> 0.9) compared to the rule of thumb (0.8), our agreement threshold permitted evaluators with low error, while rejecting one evaluator with relatively high error.

**Conclusions:**

We found that our approach established threshold values of reliability, within the context of our evaluation criteria, that were far less permissive than the typically accepted “rule of thumb” cutoff for Krippendorff’s alpha. This procedure provides a less arbitrary method for determining a reliability threshold and can be tailored to work within the context of any reliability index.

## Background

Inter-evaluator reliability is a widely-debated topic relevant to a variety of fields such as communication, computational linguistics, psychology, sociology, education, and medical science, among others [1, 2]. Although the consequences of evaluator-based tests vary, some evaluator-based tests, such as those used in medicine, may strongly influence the diagnosis and treatment of patients.

Evaluators are typically employed when a desired functional or clinically-relevant value is otherwise unmeasurable. In some of these cases, there may indeed be an objective or ideal “correct” answer, but because the variable is, in principle, unmeasurable, it is impossible to know how accurate the evaluator is in arriving at this ideal answer. By generating data using multiple evaluators and comparing responses, we can begin to gauge the quality of the evaluators, the measurement process, the generated data, the evaluators, and the resulting conclusions [3–6].

Inter-evaluator reliability is discussed using different terminologies across disciplines, with concepts such as evaluator “agreement” and “reliability” used to varying degrees of consistency. Regardless of the terminology, inter-evaluator reliability can be described as the likelihood that different influences such as evaluators, methods, and approaches, will produce the same results or interpretations [2, 7]. More formally, the keyterm ‘reliability’ is defined as the ratio of the variability of what is being measured to the variability of the measurement process [8]. Therefore, high reliability indicates that measurement error is small, while low reliability suggests high variability and measurement error. For evaluator-based tests, inter-evaluator reliability cannot be measured directly [9]. As the variable of interest is not precisely known, comparisons between its true variability and the variability of the measurements cannot be made. Instead, agreement between evaluators is measured and used as a proxy to qualitatively infer inter-evaluator reliability [9–11].

The effective usage of inter-evaluator agreement measures is limited by a lack of standardization in application and interpretation. For example, many statistics to measure inter-evaluator agreement (commonly referred to as “inter-evaluator reliability indices”) have been proposed; however, because of the specificity required by actual implementation, most are considered unsuitable for general use [5, 7, 9, 12, 13]. This means that different studies may often use different reliability indices, which may make comparisons of their results problematic. Perhaps the greater limitation with inter-evaluator reliability indices is the general difficulty in interpreting their numerical outcomes; understanding these numerical outcomes is critical to appropriately assessing the trustworthiness of the reliability data. Typically, the possible values for a reliability index range from 0 to 1, where 0 suggests the absence of reliability and 1 suggests perfect reliability. Devising a universal threshold of “acceptability” between 0 and 1, that works for any dataset independent of context, is not likely possible [4]. For most indices (e.g., Bennet et al.’s *S*, Cohen’s κ, Scott’s π, Krippendorff’s α) it is commonly suggested that a cutoff threshold value of 0.8 is a marker of good reliability, with a range of 0.667 to 0.8 allowing for tentative conclusions [4, 9, 11, 13–16]. Interestingly, these threshold values are often employed with the knowledge of their largely arbitrary determination, and used in spite of suggestions that they are likely unsuitable for generalization [4, 10, 11, 15, 16]. This can pose the problem of incorrect interpretation of results, as using an unacceptably-low agreement threshold can result in unreliable data being trusted and increasing the likelihood of drawing invalid conclusions. Inversely, an overly-strict agreement threshold may lead to discarding valid findings. An inappropriate agreement threshold could also preclude opportunities for exploring and correcting sources of unreliability in evaluators and/or evaluation methods. An ideal threshold value would be derived through analytical methods that provide a meaningful number in the specific context of its application and use.

An examination of the literature suggests that the issue of determining an appropriate reliability threshold is still an open problem, as few-to-no methodologies have been adopted for the determination of contextually-relevant threshold values to facilitate drawing conclusions from inter-evaluator data [2, 13]. Indeed, other investigators are still working to tackle this issue. Wilhelm et al. conducted a simulation study, with themes similar to those described in this paper, to determine how agreement thresholds impact the results of reliability studies [17]. The necessity for a solution to this problem is clearly evidenced by a severe lack of consistency and systematicity in how inter-evaluator reliability measures are interpreted. In fact, we examined seven clinically-relevant inter-evaluator reliability studies that have been published since 2015 and found that for 4 of the 7 studies, it was unclear how benchmarks of reliability were determined (i.e., what constituted a good versus bad score) [18–21]. The three remaining studies each used a different source for inter-evaluator reliability interpretation guidelines, and thus used slightly different grading scales [22–24]. Additionally, reliability indices alone cannot tell us the error inherent to a group of evaluators attempting to measure a variable of interest. For example, Wilhelm et al. reviewed articles in two major journals and found that researchers tended to report inter-rater agreement above 0.80, without addressing the magnitude of score differences between raters [17], which is a central theme of our paper.

In addressing these fundamental gaps, this work develops a methodological framework to bridge the concepts of inter-evaluator reliability (reliability indices) and the potential measurement error in a functional or clinically-relevant value. We illustrate and evaluate the application of this framework using a quantitative example, where evaluators extracted time intervals from specific cues in video footage. We suggest that using this methodology, application-specific reliability thresholds can be determined for most any given task or reliability index. The development of such a technique may help unlock acceptable reliability index thresholds, establish performance benchmarks for evaluator training programs, and provide context directly to applications of reliability indices.

## Methods

The methods are presented as a general methods section and a quantitative example. The quantitative example illustrates the application of the general methodology, and evaluates the performance of the techniques presented. It is important to note that the specific measures used in the quantitative example (i.e., Krippendorff’s alpha and percent error) were chosen for our specific application, and the general methods described in this paper are not limited to these reliability and error measures.

### General methods

**Fig. 1:**
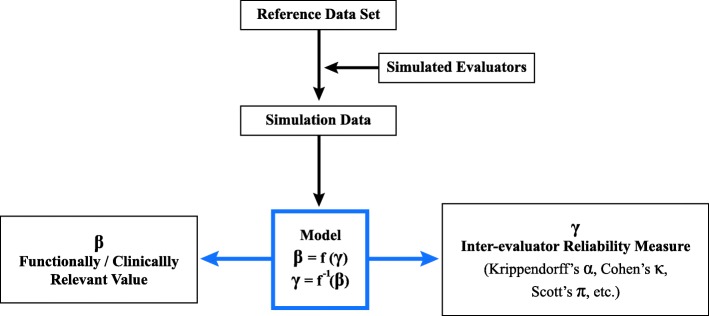
The methodological framework to bridge inter-evaluator reliability measures and measurement error in functionally or clinically-relevant values. Where β and γ represent a chosen value of interest and reliability measure, respectively

The goal of this work is to develop a methodology to establish a relationship between a chosen reliability index and the measurement error of a functional or clinically-relevant value. Our approach involves generating a large population of simulated evaluator data, and then calculating the error and agreement of each against a reference dataset. This creates a model between evaluator agreement and error, that describes how much error could be expected based on any given level of agreement (Fig. 1). The below step-by-step approach describes how this method is generally applied. The instructions are intended for investigators with a modest background in mathematics, statistics, and basic programming (such as MATLAB). The simulation time will vary based on several factors (calculation optimization, dataset size, simulation iterations, etc.). For our quantitative example, the simulation and calculations took approximately 1 to 2 h to run on a standard office desktop computer.

#### Establish a reference dataset

A reference dataset must be established for the evaluator test that is being modelled. The only requirement for the reference dataset is that it is representative of a typical dataset from that test. The reference dataset is *not required* to be empirical data; rather, the reference dataset could be generated from a *distribution of*, or a *distribution parameterized to resemble*, a population of test scores. This reference dataset will be used as a basis for generating and comparing the simulated evaluator population. There are no specific requirements for the length of the reference dataset. The authors do not wish to conjecture on the appropriate amount of data to be used when working with reliability indices, as the answer is likely context-sensitive, and has been investigated by others [25, 26]. At a minimum, it would seem necessary to include at least the same amount of data that would be used in a practical application or research study using that reliability index. For example, if one were generating a model to provide context to field applications of inter-evaluator reliability measures, then using a reference dataset of the same size that has been determined appropriate for those field applications would likely provide the most relevant model. It should be noted that the reference dataset is *not required* to be single instance of an evaluator test, but instead could be a concatenation of results from multiple independent instances of that test. Mathematically, we will refer to the reference dataset as *I*_*m*_, where *m* is the number of measurements in the dataset.

#### Simulate a population of evaluators

For purposes of simplification, the reference dataset can now be thought of as a “perfect evaluator.” That is, the reference dataset is considered to be the result of an evaluator who is perfectly reliable, and has obtained the “correct” answer from evaluating the test. As stated above, the reference data do not need to be correct (or even empirical data), they are only referred to as correct for purposes of generating the model because they are used as a basis of comparison. Their actual correctness has no bearing on the model’s accuracy or validity. Each new evaluator is simulated by taking the reference dataset and introducing Gaussian noise into it. Taylor has shown that a Gaussian distribution is a valid first-order approximation of the two observational errors that are inherent to any system of measurement: systematic errors and random errors [27]. These errors can be modelled by modifying the first (mean) and second (standard deviation) moments of the Gaussian distribution, respectively [27]. In essence, the Gaussian distribution can be thought of as an evaluator whose likelihood of making a systematic error or random error is described by the mean and standard deviation of the Gaussian distribution. For example, an evaluator who makes systematic errors is described by a Gaussian distribution with a shifted mean (errors are systematically in the same direction) whereas an evaluator who makes random errors is described by a Gaussian distribution with a large standard deviation (errors randomly fall on either side of the average measurement). In practice, evaluator mistakes may not be perfectly Gaussian, but over many simulated trials, all evaluator errors would tend to a Gaussian distribution due to the central limit theorem.

A set of random variables from Gaussian distributions (we will call this set “$\mathcal{X}$”) must be generated to capture *at least* the range of evaluator behavior that may be practically expected; additional distributions may be generated to capture more erroneous behavior, but this may incur additional computation time. The total number of random Gaussian variables (the size of set $\mathcal{X}$) generated will depend on the chosen step-size and range of systematic error and random error to be investigated, and this is heavily dependent on the specific task. In many cases, it may be appropriate to increment systematic error and random error by the resolution of rating units used in the test (i.e., the smallest change in measurement an evaluator can make). If a continuous scale is being used, an appropriate error step-size will have to be determined; there is no set procedure for determining the error step-size, and this will have to be done at the discretion of the investigator. A general rule might be to use the smallest step-size that is still detectable or has meaningful relevance to the test (e.g., a step-size of one nanosecond would be too fine of a resolution for a human reaction time task, whereas a step-size of a second would be too coarse). In the case of nominal data, where only two rating units are available, the error step-size may be thought of as a probability step-size. It should be noted that this method allows for the step-size and range of systematic error and random error to be chosen independently.

The chosen values of systematic error to investigate will be represented by the array *μ*, and the length of that array (the chosen range of error divided by the chosen resolution) will be referred to as, and indexed by, *i*. Similarly, the values of random error will be represented by the array *σ*, and the length of that array will be referred to as, and indexed by, *j*. Additionally, the *μ* and *σ* arrays should each contain the value “0,” representing an evaluator who does not make that type of measurement error. Therefore, the equation

1 $$ {\mathcal{X}}_{ij}\sim \mathcal{N}\left({\mu}_i,{\sigma}_j\right) $$

describes an *i* by *j* matrix of Gaussian random variables, where *i* and *j* index the mean (*μ*) and standard deviation (*σ*) of the ${\mathcal{X}}_{ij}$ Gaussian distributions from which the random variables were generated, respectively. New i * j Gaussian random variables are generated along the *m* dimension, so that there are *i* by *j* random variables for each *m* measurement in the reference dataset. Next, the reference dataset, *I*_*m*_, must be replicated along the *i* and *j* dimensions. Thus, *I*_*ijm*_ is a matrix of the reference data and there are *i* * *j* copies of each *m* measurement, so that each *m* measurement can be modified by each *ij* Gaussian random variable. The final step is to generate the simulated evaluator population, *I′*, calculated as

2 $$ {I}_{ijm}^{\prime }={I}_{ijm}+{\mathcal{X}}_{ijm}. $$

Each element of *I*_*ijm*_ is modified by a random value generated from one of the Gaussian distributions, whose parameters are described by *i* and *j*. The result is a population of simulated evaluators, matrix *I’*_*ijm*_. Each *ij* evaluator, whose measurement errors are described by ${\mathcal{X}}_{ij}$, has made *m* measurements. As previously stated, the evaluator whose *μ* = 0 and *σ* = 0 is the perfect evaluator (the initial reference dataset) who will be the basis of comparison for all other simulated evaluators.

This process relies on the sampling of random variables (${\mathcal{X}}_{ijm}$), and therefore the simulation should be repeated *N* multiple times. This smooths the randomness of the data and allows for a more robust model. Our methodology has no inherent requirements about the number of times the simulation should be repeated. Many others have investigated optimal sample sizes for simulation studies [28, 29], and their work may be considered when choosing the number of iterations for this simulation method. In general, increasing the number of simulations improves the final result but increases computation time. Thus, the result of the simulation should be *i* * *j* evaluator datasets of length *m*, where each *ijm* combination has been simulated *N* times (with each simulation sampling *ijm* new random variables from the *ij* Gaussian distributions).

#### Create the model

Once the evaluator population has been simulated, agreement and error must be calculated for each of the N * i * j evaluators. In other words, each combination of *i* and *j* must be compared to the *μ* = 0, *σ* = 0 perfect evaluator (the reference dataset). Our method is not limited to any particular agreement or error calculation, so this step is dependent on the reliability index and error calculation that are most appropriate to the evaluator task. However, the manner in which agreement and error measures are calculated should be reflective of how they would be applied in practice. For example, if the result of an evaluator test is interpreted by *adding* all of the evaluator’s individual scores together, then error should be calculated on this *sum*. Alternatively, if an evaluator’s results are interpreted by *averaging* all of their individual scores together, then error should be calculated on this *averaged* score. Generally, it is likely that the best method for calculating agreement is to compare each individual score between the evaluators. However, it is most important that agreement is calculated in the way that has been deemed most appropriate for the practical application or research study, as this will provide the most relevant model between agreement and error.

Once agreement and error have been calculated for each evaluator relative to the reference dataset, agreement and error should be averaged by observational error parameters across all *N* simulations. That is, every evaluator who had both the same *μ* and *σ* could be considered to have been the same evaluator (as they were exactly as probable to make the same mistakes), and therefore their agreement and error from all *N* simulations should be averaged together to quantify their average performance, thus smoothing the simulated data. This should result in i * j four-dimensional datasets which each contain an agreement, error, μ_i_, and *σ*_*j*_ value, one for each i * j evaluator. The agreement and error of all evaluators can now be plotted against each other to model how much error can be expected from an evaluator based on their level of agreement. Optionally, systematic error (*μ*_*i*_) and random error (*σ*_*j*_) can be plotted for each evaluator (e.g., by color or size of markers, see below for example) to further understand how observational errors affect agreement and error. It would generally be expected that an envelope would form; essentially, this is a boundary that emerges which describes the most amount of error (worst-case error) an evaluator could be expected to have, based on their calculated agreement. We demonstrate this below in our quantitative example. A function may be fit to this envelope which allows a mathematical description relating agreement and worst-case error.

### Quantitative example

This quantitative example is provided to illustrate how the method is applied, and because it was a practical challenge that we encountered in our research; the solution to which was the basis of this general method. Again, it is important to clarify that the specifics of how the method is applied (agreement measure, error measure, etc.) to this evaluator task are not due to inherent limitations of the method, rather, our selected reliability index, error measure, error-step size, etc., were chosen as the most appropriate for our particular evaluator task.

#### Establish a reference dataset

Three research professionals analyzed video footage, with the goal of improving internal processes. They judged and recorded times of two distinct reoccurring events. The video was obtained under a Defense Advanced Research Projects Administration research study and contained footage of an anonymous consented participant transferring rubber blocks between two compartments of a wooden box. Evaluators worked in isolation using media player software which allowed forward and backward frame-by-frame scrubbing. They scanned through the video and determined the times at which the participant grasped blocks in one compartment and the times at which they released them into the other compartment. They recorded these video timing events into a spreadsheet, producing a total of 300 timing measurements for each evaluator. Each individual timing event was taken and averaged across the three expert evaluators to produce a single representative 300-point reference dataset.

#### Simulate a population of evaluators

We wrote a custom MATLAB (Mathworks, Natick, MA) script to create 49 unique simulated evaluators by injecting error into the reference dataset. To do this, the script generated 49 Gaussian distributions, each with different combinations of mean and standard deviation parameters, which represented systematic error (*μ*) and random error (*σ*), respectively. In other words, each of the 49 combinations of *μ* and *σ* generated a distinct Gaussian distribution, where each Gaussian distribution represented the observational errors made by different simulated evaluators. The analyzed video footage was recorded at 30 frames per second, so the step-size for *μ* and *σ* of the Gaussian distributions was chosen to be 0.033 s (one video frame), the smallest possible error. The values of *μ* and *σ* ranged from 0 to 0.198 s (0 to 6 video frames), for a total of 49 combinations. In cases where *μ* was low and *σ* was high, some simulated evaluators occasionally selected negative values (about 0.3% of measurements). While our chosen range of random error resulted in this unrealistic circumstance, we wanted to ensure that we captured a sufficient range of evaluator error to build our model. In our application, negative and zero-duration timing events were not possible, so these values were defaulted to 0.033 s (the minimum resolvable time for an event, one video frame). This introduced a small amount of bias to cases with high random error relative to systematic error which can be seen in the results; we discuss this in more detail below.

Three hundred random numbers were then chosen from each of the 49 Gaussian distributions and added to the reference dataset to create 49 new unique hypothetical datasets each containing 300 modified timing values, as described above in Eqs. (1) and (2). In other words, each simulated evaluator made 300 different “mistakes”, one for each of the 300 measurements, with each error drawn from the evaluator’s own Gaussian distribution. Each of the 49 modified datasets represented a set of 300 imperfect scores generated by a simulated evaluator. It should be noted that our choice of using 300 measurements reflects how we would use inter-evaluator agreement in a practical application. That is, a complete execution of the test produces 300 measurements, and we would ideally measure inter-evaluator agreement on a full test dataset; thus, we chose to generate our model using a full 300-measurement dataset.

To smooth the randomness of the data, we repeated this process 100 times for each of the 49 Gaussian distributions. We stopped the simulation after 100 iterations, as this produced a smooth monotonic relationship between increasing random error and decreasing inter-evaluator agreement, which we deemed appropriate for our specific application. This resulted in 4900 unique hypothetical datasets. These new hypothetical datasets were then used as the “results” obtained from 49 simulated evaluators, each completing the video analysis task 100 times.

#### Create the model

The Krippendorff’s alpha and percent error were then calculated for each of the 4900 datasets, pairwise with the reference dataset, to explore relationships between Krippendorff’s alpha, percent error, systematic error, and random error. We specifically chose Krippendorff’s alpha as it was appropriate in the application of our data, which were time measurements (ratio data type). Krippendorff explains in detail how Krippendorff’s alpha is calculated in his 2011 manuscript [30]. Here, we briefly summarize the calculation. We wrote a custom LabVIEW (National Instruments, Austin, TX) program which used the coincidence matrix calculation method (Eq. 3),

3 $$ \alpha =1-\left(n-1\right)\frac{\sum_c{\sum}_k\kern0.5em {o}_{ck}\ast \kern0.5em {{}_{metric}\delta}_{ck}^2}{\sum_c\sum \limits_k\kern0.50em {n}_c\ast {n}_k\ast {}_{metric}{\delta}_{ck}^2}, $$

and verified the accuracy of our program with ReCal OIR [30, 31]. Equation 3 was used for all Krippendorff’s alpha calculations, where *n* is the total number of measurements collected, *c* and *k* are each a separate index into the same set of unique values that increment independently to allow for generation of every allowable pairwise combination. The allowable pairwise combinations are *x*_*c*_ and *x*_*k*_ (the reliability data, see Eq. 4) for all possible values of *c* and *k* whereas *n*_*c*_ and *n*_*k*_ are the number of times that *x*_*c*_ and *x*_*k*_ are used in total. The number of occurrences of *x*_*c*_ and *x*_*k*_ value pairings within the reliability data are represented by *o*_*ck*_. The type of reliability data being used dictates which “difference function” to use, defined as ${{}_{metric}\delta}_{ck}^2$, where *metric* is the data type. The difference function for ratio data is shown in Eq. (4):

4 $$ {{}_{ratio}\delta}_{ck}^2={\left(\frac{x_c-{x}_k}{x_c+{x}_k}\right)}^2. $$

**Fig. 2:**
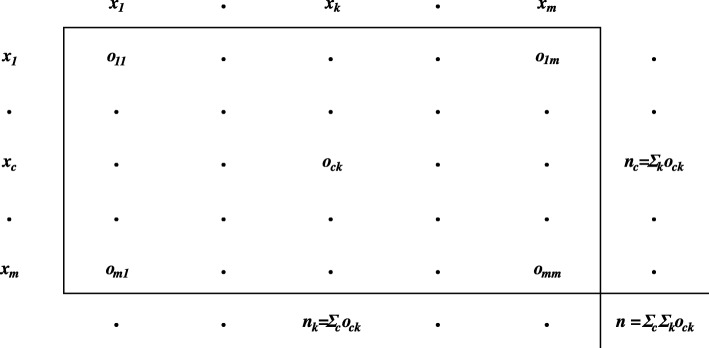
The general form of a coincidence matrix used for calculating Krippendorff’s alpha. The matrix tabulates all the unique values from the data and the number of times those values were assigned to a common item by different evaluators

We used the coincidence matrix to calculate Krippendorff’s alpha because it is the most computationally efficient method (Fig. 2). For our percent error calculations (Eq. 5, where *Theoretical* is the reference measurement and *Experimental* is the simulated measurement),

5 $$ Percent\ error=\frac{\left| Theoretical- Experimental\right|}{Theoretical}\ast 100, $$

we decided what part of the task provided the highest level of error. In our video rating task, the “speed” phase (50 timing events out of the 300) where the participant rapidly moved the blocks from one side of the box to the other was the most sensitive to timing errors because the events were so short. A small error in measurement produced a relatively large percent error. In our task, the individual errors were not important. We were interested in the total error of the 50 timing events from the speed phase, so we quantified the percent error of total time of those events. Using the error measurements of this subset provided the worst-case baseline to sufficiently capture the percent error for the most difficult aspects of the task. Also, we were not concerned with the direction of the error (i.e., whether it was negative or positive, relative to the reference value), only the magnitude of the error, so absolute value of percent error was used. It is important to understand that this described procedure for calculating error is not inherent to the methodology; it is an idiosyncrasy of the task that we are using to demonstrate the methodology. For another task, it may be more appropriate to use total error, root mean squared error, or some other calculation to quantify the error of an evaluator. We constructed the model for this task using the Krippendorff’s alpha and percent error calculations.

### Model assessment

We wanted to verify that our model accurately described the relationship between the agreement and error of actual human evaluators, and also demonstrate the applicability of the model in a real-world scenario. We first fit a curve (y = − 0.637x^1.76^ + 1, r^2^ = 0.999; the curve was created using Igor Pro v 6.36 Curve Fitting function; WaveMetrics, Portland, OR) to the model, using the data points of simulated evaluators who defined the envelope, to mathematically define what would be the “worst-case” percent error for any given Krippendorff’s alpha value (see Results). A power law was chosen for the curve fit as it provided a high r^2^ for the envelope, over the range of data that we were interested in. This is, again, a facet of the methodology that is dependent on the evaluator task; our methods are generalizable to allow any form of curve fitting. We then compared the results of the hypothetical modeling to an actual population of evaluators to demonstrate that the model values reflect the values actually generated by human evaluators. To do this, we assigned the video analysis task to a new cohort of trained human evaluators (*n* = 5). We used the reference dataset from our panel of expert evaluators (described above) as a standard of comparison and calculated Krippendorff’s alpha and percent error values for each of the human evaluators to verify their compliance to the model.

Since the test data that were used to generate the model were also the same data that the evaluators were scoring, it was a concern that any comparisons between the evaluators and the model were circular and not generalizable to new instances of the test. To explore this idea we repeated all of the simulation steps using a new scoring video of a different anonymous participant taking the same test, scored by a new evaluator. All error step-sizes, error calculations, and agreement calculations were methodologically identical to the original simulation described in “Quantitative example”. We again plotted the results of the human evaluators from the *original* video analysis, to see if they were still well-explained by the new model, as well as fit a curve to the new envelope (y = − 0.6251x^1.685^ + 1.001, r^2^ = 0.9998) to determine if there was any change between the two models in the mathematical relationship between agreement and error.

## Results

**Fig. 3:**
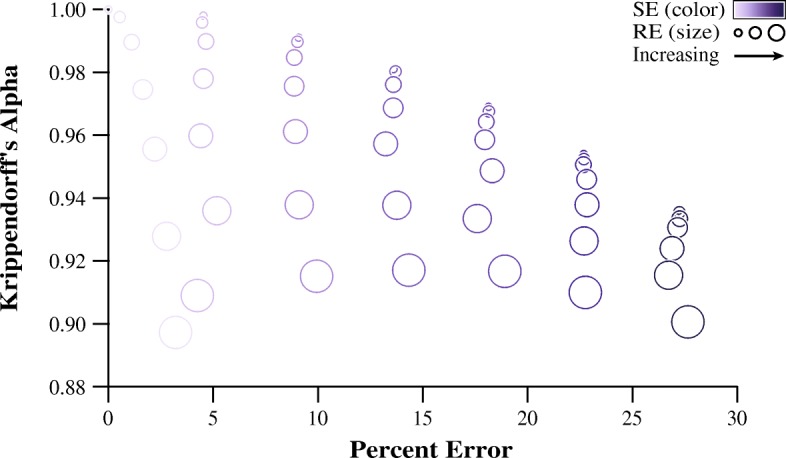
This scatterplot shows the average Krippendorff’s alpha and percent error values for each of the 49 simulated evaluators, with the size and shading of the markers representing random error and systematic error, respectively. An envelope exists which describes the upper-bound of percent error for any given value of Krippendorff’s alpha for this video analysis task

The Krippendorff’s alpha values for the 4900 hypothetical datasets ranged from 0.998 to 0.854 and the percent error values ranged from 0 to 39.4%. Figure 3 shows the average Krippendorff’s alpha and average percent error of all 49 (averaged) simulated evaluators, with the size and shading of the markers representing the random error and systematic error parameters, respectively. For our evaluator task an increase in systematic error was correlated to an increase in percent error (Pearson’s *r* = 0.997, *p* < 0.001), whereas random zero-mean error did not correlate to an increase percent error (Pearson’s *r* = − 0.025, *p* = 0.865). This was a result of our task being specifically concerned with average percent error; simulated evaluators with zero-mean random error produced modest percent error when averaged over many trials. As seen in Fig. 3, this result was also evidenced by darker circles falling farther to the right with larger circles of the same color aligning vertically. As mentioned above, flooring our measurements to 0.033 s introduced a small amount of systematic error into simulated evaluators who were prescribed low systematic error and high random error. This can be seen in Fig. 3 as the markers with increasing random error are staggered to the right when systematic error is low. This deviance was not a concern for our specific investigation as we were only interested in the envelope of the model.

### Model assessment

**Fig. 4:**
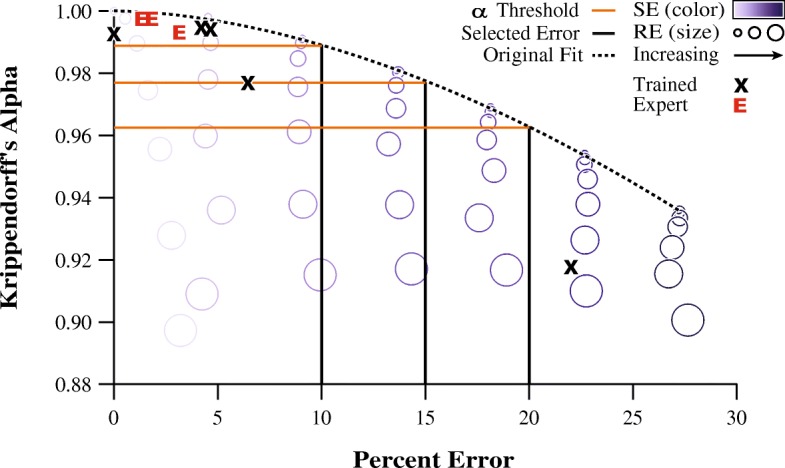
This scatterplot shows the average Krippendorff’s alpha and percent error values for each of the 49 simulated evaluators. The curve was fit to data points of simulated evaluators with systematic error only as these points provide the worst-case percent error for a given value of Krippendorff’s alpha. The results of the expert human evaluators are shown as “E’s” and trained evaluators are shown as “X’s”. The darker vertical lines represent theoretical levels of percent error and the lighter horizontal lines are corresponding Krippendorff’s alpha thresholds for enforcing those error limits. This demonstrates how a Krippendorff’s alpha threshold can be used to limit the amount of error from evaluators based on the observed relationship from the model

To verify that our model accurately described the evaluator agreement and measurement error relationship of actual human evaluators, we plotted the Krippendorff’s alpha and percent error values collected from our human evaluators against the performance of the simulated evaluators. We found that the results of the human evaluators fell within the envelope defined by the simulated evaluator performance (Fig. 4).

**Fig. 5:**
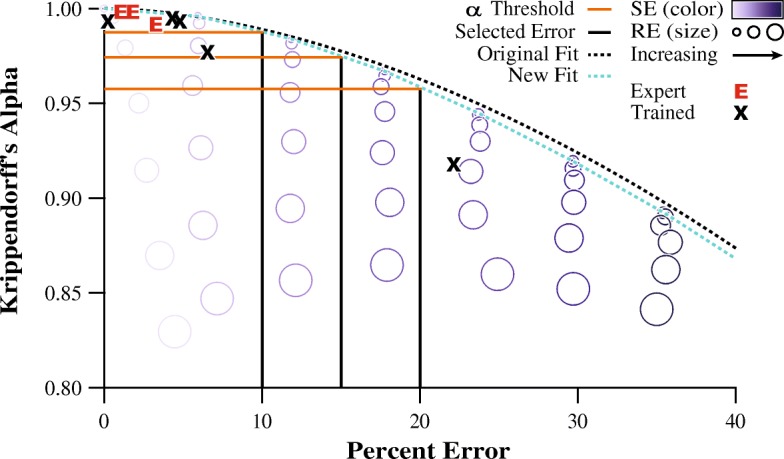
This model was generated from the results of a trained evaluator analyzing a separate instance of the block moving task and used to gauge the performance of the human evaluators on the original video analysis task. The model is similar to the original. This suggests that a single model is suitable for generalization to other instances of this video analysis task and model generation only requires a typical dataset

Figure 5 shows the model created using the results of the new trained evaluator analyzing this new instance of the block moving task. The original fit from the model in Fig. 4 still provides an accurate description of the Krippendorff’s alpha and percent error relationship (r^2^ = 0.991). The lighter dotted line is the new fit (y = − 0.6251x^1.685^ + 1.001, r^2^ = 0.9998) and is provided for comparison. The two models are highly similar, and are nearly identical when considering percent error of 10% or less. Both models show high agreement and low error for the tightly clustered six evaluators, who would pass any of the example Krippendorff’s alpha thresholds presented, and one evaluator who has relatively low agreement and excessively high error that would not pass any of the thresholds.

## Discussion

When measuring agreement between evaluators, a decision about what constitutes an acceptable level of agreement must be made. Historically, interpreting an agreement measure was ambiguous, as the practical implications of choosing one threshold over another were not well-defined. This led to general use of a 0.8 “rule of thumb” value as a threshold, though several works have suggested that this cut-off is not likely suitable for all studies [4, 10, 13–16]. To address this issue, we developed a systematic approach to arrive at a relevant context-specific reliability threshold, bridging the gap between reliability indices and the error inherent to the test construct of interest. Our approach simulated the results of a large population of evaluators. In our quantitative example, these simulated evaluators “judged” a video analysis task. Our method injected known tendencies for making systematic and random errors, and calculated the agreement (Krippendorff’s alpha in our example) and the error (percent error in our example) between the simulated data and a reference dataset. This procedure allowed us to relate agreement, which is customarily measured in reliability studies, to the amount of error from the “true” values, which is more salient but typically unavailable. In our example, we found that an envelope existed which defined the maximum observed percent error for any given value of Krippendorff’s alpha. We characterized this envelope and determined its effectiveness and generalizability.

**Fig. 6:**
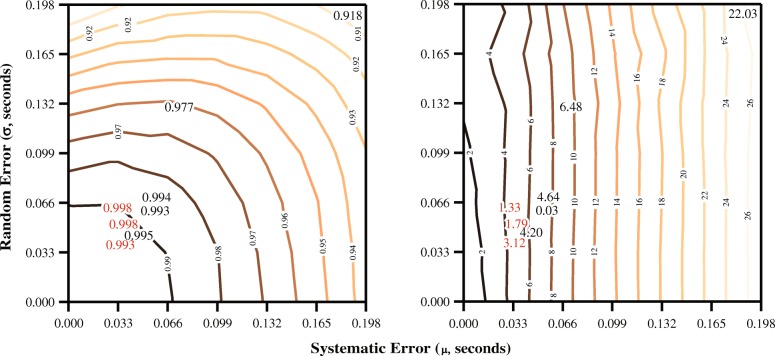
The contour plots show the Krippendorff’s alpha (left) and percent error (right) values for the simulated evaluators, for the prescribed grid of systematic error and random error. The values for the human evaluators are plotted in bold numerals (experts in lighter font), with their approximate position corresponding to their calculated systematic error and random error compared to the reference dataset. This figure provides insight into the types of mistakes being made by evaluators. Whereas the evaluators were positioned in this figure based on their calculated systematic error and random error, positioning them based on the Krippendorff’s alpha and percent error contours would still provide reasonably accurate estimates of the true amount of systematic and random error

During the evaluation of our quantitative example, we found that the results of the human evaluators adhered to the derived threshold envelope and were similar to those obtained from the simulated data (Fig. 4). As evidenced through this quantitative example, these findings support that our proposed techniques have the power to facilitate meaningful interpretations of reliability indices in a relevant context of measurement error. An additional characteristic of our quantitative example is highlighted in Fig. 6. Here, the contour plots, generated using the simulated datasets, show Krippendorff’s alpha (left) and percent error (right) values for the investigated combinations of systematic error and random error. It was demonstrated that an increase in either systematic error or random error can lead to a decrease in agreement (lower Krippendorff’s alpha), whereas percent error (the “functional value”), on average, is only affected by systematic error. This is due to the mathematical nature of random observational errors (and thus how they were modeled), as they are described by symmetrical deviations with no net change from the mean of a Gaussian distribution; therefore, they average to zero over many trials [27]. This contour plot format can be more generally applied to other reliability indices or measures of errors to illustrate the consequences of observational errors.

Finally, the data highlighted in Fig. 5 were generated from a new participant being scored by a new evaluator to verify the generalizability of our techniques. The only observed difference between this newly generated model and the original was that the contour plots (not shown) of Krippendorff’s alpha and percent error were compressed, as the values from the second test were, on average, numerically smaller than the first test. This means that systematic error and random error had relatively greater effects on Krippendorff’s alpha and percent error. Regardless, the Krippendorff’s alpha and percent error relationship generated by this model remained a valid and useful way to assess evaluator reliability in this video analysis task. This was evidenced by re-plotting the human evaluators on the new model, as they fell within similar error and agreement thresholds as the original model (Fig. 5). These data suggest that once a relationship between a selected reliability index and functional measure has been established, that result is generally applicable to any instance of that same test, scored by any cohort of evaluators, without need to revisit the model.

This example demonstrates the use of our systematic procedure to both investigate the consequences of different agreement thresholds and provide a framework for researchers to make informed decisions about reliability in their evaluator-based tests. Modeling a large population of evaluators with a variety of prescribed probabilities for making mistakes and then calculating their resulting error allowed us to describe our chosen agreement measure in the context of how the data would be used practically. In our example, we generated a standard of comparison for this specific instance of our test by employing a group of expert evaluators. Comparing the experts’ results to the trained evaluators’ results revealed practicable levels of agreement and the error associated with that agreement. We found that high levels of agreement (0.99 and up) were regularly achieved and afforded error of no greater than 5%. Using our model as a frame of reference we concluded that a Krippendorff’s alpha threshold of 0.985 should be used for this task to permit error no greater than 12% while not being so strict as to potentially throw out useful data. It is critical to note that the conventional 0.8 rule of thumb threshold would have been egregiously permissive in our quantitative example, further reinforcing the need for application-specific agreement thresholds.

A key strength of our methodology is its high level of customizability which greatly expands its scope and utility. This procedure should be applicable to most any agreement measure or evaluator-based task, and may be uniquely tailored to emphasize the important aspects of the task or to reflect how the data will be used in practice. The investigator may choose the step-size of the simulated evaluators’ errors and the approach for calculating error (using percent error or a different error calculation entirely, calculating error on individual values or averages values, etc.) beforehand, based on the specific needs of that test. Additionally, the investigator is able to decide how the “true” or reference data that are used for calculating agreement and error are defined or generated. It should be noted that the presence of true or even presumably-true values are not necessary for using this method, and the values used to generate the model are not even required to be actual results from the specific test of interest. The only requirement for these reference data is that they are representative of the types of values resulting from an evaluator judging the specific test. In our quantitative example, the error step-size was chosen to be the resolution of the measurements of the test (based on the video frame rate). We used percent error calculations from a subset of the measurements which represented a particular metric of interest. We determined this metric to be most sensitive to error so using it for our calculations provided us with a worst-case percent error for any given level of agreement. The “true” or reference values were established by averaging together the results of the expert evaluators.

Our methodology may provide a useful framework for establishing agreement benchmarks in evaluator-based tests, and could be adapted for application in other contexts. For example, the reliability of clinical tests which require human evaluation is a major concern. The accuracy and validity of these types of assessments could be improved by using our methodology. To implement our methods more generally into a clinical context (or other evaluator-based applications), a possible approach would be first building a “true” or reference set of measurements for a typical application of the test. A baseline panel of expert evaluators could be employed to generate the reference measurement(s) for that particular application. Modeling a simulated evaluator population from this dataset would establish an agreement-error relationship that could be used as a “grading scale,” to relate evaluator quality (inter-evaluator reliability) and measurement error, that would generally apply in any instance of that test. This process would only need to be performed once, and the resulting scale could then be incorporated into training programs for new evaluators or used to periodically assess groups of existing evaluators as a measure of quality control.

Perhaps the biggest limitation of this work is that the models generated are inherently more accurate when the reference data are of similar numerical magnitude to the data typically obtained from the testing procedure. Although a “one size fits all” solution would be ideal, multiple models may be necessary if the results of the evaluator task vary greatly. For instance, in our quantitative example, video footage of a healthy participant transferring objects with their upper limbs was scored. If this task were performed with a sensorimotor-impaired participant, where scores would be anticipated to differ significantly from healthy performance, it may be necessary to generate a new model built from reference data that are more reflective of the anticipated sensorimotor-impaired results. Further work could be done to investigate the consistency of models over different ranges of numerical values and how, and to what extent, the models diverge from the data. This methodology could potentially reveal strengths and weaknesses of different reliability indices and possibly inform the selection of an appropriate agreement measure. Our approach of using zero-mean random error may present as a limitation as it is an idealized circumstance. This zero-mean approach could perhaps be replaced with a more nuanced approach exploring the skew and kurtosis of error distributions to potentially reveal additional findings. Skew and kurtosis may have the potential to model more peculiar erroneous evaluator behavior, such as heavy-tailed outlier data, which could be approximated by increased kurtosis. A particularly troublesome example would be an evaluator who occasionally selects extreme values in an asymmetric fashion to intentionally bias the outcome of an evaluation. Our methodology could be used to model what this behavior looks like from a reliability perspective, to help detect and mitigate this type of behavior in the field.

This work aims to provide a generalizable procedure, yet datasets in evaluator-based scoring activities may be diverse in size, variability, and data type. Thus, it is not feasible to devise a universal procedure which can accommodate all possible variants of reliability data. As such, each individual application of this methodology requires the discretion of the investigator. Furthermore, this method could reasonably apply to many data types (e.g., nominal, interval, ratio), error measurements (e.g., percent error, RMS error, mean absolute error), and reliability indices (e.g., Cohen’s κ, Scott’s π, Krippendorff’s α). We suggest that the quantitative basis of this method represents an improvement over rule of thumb conventions for interpreting reliability indices.

## Conclusion

By simulating a population of evaluators with predetermined probabilities for making mistakes, we have explored correlations between evaluator reliability indices and functional test values of interest. We demonstrate this method using a quantitative example to derive a relationship between Krippendorff’s alpha and percent error. Through this simulation and modeling we assessed the quality of our human evaluators based on their alpha coefficients. We propose that this is a reasonable technique for establishing agreement thresholds to identify suitable evaluators and this technique could be expanded for use in other evaluator-based tests, or with different agreement and/or error measurements.
